# Reaping what we sow: Centering values in food systems transformations research

**DOI:** 10.1007/s13280-024-02086-5

**Published:** 2024-11-16

**Authors:** O. Care, Julie G. Zaehringer, Michael J. Bernstein, Mollie Chapman, Cecilie Friis, Sonia Graham, L. Jamila Haider, Mónica Hernández-Morcillo, Harry Hoffmann, Maria Lee Kernecker, Hannah Pitt, Verena Seufert

**Affiliations:** 1 Stockholm Resilience Centre, Stockholm University, Albanovägen 28, 106 91 Stockholm, Sweden; 2 Wyss Academy for Nature, Kochergasse 4, 3012 Bern, Switzerland; 3 AIT, Austrian Institute of Technology, Gieffengasse 4, 1210 Vienna, Austria; 4 Transdisciplinarity Lab, Department of Environmental Systems Science, ETH Zurich, Universitaetstrasse 22, 8092 Zurich, Switzerland; 5 Department of Geosciences and Natural Resource Management, University of Copenhagen, Øster Voldgade 10, 1350 Copenhagen, Denmark; 6 Australian Centre for Culture, Environment, Society and Space, University of Wollongong, Northfields Ave, Wollongong, NSW 2220 Australia; 7 University for Sustainable Development, Alfred-Möller-Str. 1-Haus 11, 16225 Eberswalde, Germany; 8 TMG Research gGmbH, EUREF Campus 6-9, 10829 Berlin, Germany; 9 ZALF, Eberswalder Str. 84, 15374 Müncheberg, Germany; 10 Cardiff University, Glamorgan Building, King Edward VII Avenue, Cardiff, CF10 3WA Wales, UK; 11 Institute of Social Sciences in Agriculture, Department Sustainable Use of Natural Resources (430C), University of Hohenheim, Stuttgart, Germany; 12 Centre for Development and Environment, University of Bern, Mittelstrasse 43, 3012 Bern, Switzerland; 13 Institute of Geography, University of Bern, Hallerstrasse 12, 3012 Bern, Switzerland; 14 Centre for Development and Environment, University of Bern, Mittelstrasse 43, 3012 Bern, Switzerland; 15 Institute of Geography, University of Bern, Hallerstrasse 12, 3012 Bern, Switzerland

**Keywords:** Care, Collectivity, Process work, Reflexivity, Sustainability research, Transformational leadership

## Abstract

In many transdisciplinary research settings, a lack of attention to the values underpinning project aims can inhibit stakeholder engagement and ultimately slow or undermine project outcomes. As a research collective (The Careoperative), we have developed a set of four shared values through a facilitated visioning process, as central to the way we work together: care, reflexivity, inclusivity, and collectivity. In this paper, we explore the implications of a values-centered approach to collaboration in food system transformation research. The paper presents two cases that illustrate how researchers might approach centering values in practice. Where much research on food system transformation focuses on values of food system stakeholders, we contribute insights into the values of researchers in such transdisciplinary endeavors. Specifically, we argue that researchers working on sustainability transformations need to be better prepared to engage in such reflections and aspire to embody values aligned with the transformations they seek to research.

## Introduction

Sustainability transformations in food systems are inherently values-led. Yet frequently in policy and research, the values behind such processes remain unexamined and implicit. Without bringing values to the fore, transformation-seeking processes can stumble. This was seen in a backlash to the 2021 UN Food systems Summit (UNFSS) and the respective UNFSS stock-taking moment in 2023 (UN Secretary-General Call to Action (unfoodsystemshub.org)), where a coalition of academics and civil society actors outside the summit called for greater attention to the values and means of participation in global food system governance (Canfield et al. 2021). One key complaint was that despite claims of inclusivity, the summit's participant list was dominated by corporate invitees (Fakhri 2022). In many transdisciplinary research settings, effort is invested in developing shared research questions and determining how diverse knowledges will be drawn together (e.g., Defila and Di Giulio 2015). As in other collective endeavors, a focus on shared goals without consideration of values can inhibit stakeholder engagement and ultimately slow or undermine project outcomes (Bagavathiannan et al. 2019). How then, can we as researchers more explicitly consider the values embodied in our research and the ways that we work together—with other academics and in transdisciplinary research?

There is a growing body of research on values within sustainability science (Kenter et al. 2019; e.g., Raymond et al. 2019; Arias-Arévalo et al. 2023), as well as the biodiversity conservation literature, recently synthesized in the IPBES Values Assessment and an associated special issue (Pascual et al. 2023). Much of this work focuses on the importance of acknowledging diverse or plural values, which especially go beyond the dominance of instrumental or monetary values. It points to the need to make space for more inclusive research and policy, attending to issues of power and justice (Arias-Arévalo et al. 2023; Kelemen et al. 2023; Lenzi et al. 2023; Pascual et al. 2023). This includes research on the values of different stakeholders or groups (e.g., Estévez et al. 2015), how to integrate these values into decision-making, policy (e.g., Bullock 2020), and transdisciplinary research (e.g., Kenter et al. 2019), and even how values can function as deep leverage points for transformation (e.g., Abson et al. 2017; Horcea-Milcu 2022). Specific to food systems is also a large body of literature on the values of different food system actors, such as farmers or consumers, including an entire journal on the topic: Agriculture and Human Values.

These various traditions encompass research on the relationship between values, motivation and behavior, the use of values in deliberative decision-making or stakeholder negotiations, as well as research on what values are and how they change or resist change. However, rarely does research on values within biodiversity, sustainability, or food systems literature focus on the values of researchers themselves. Certainly, topics such as reflexivity or positionality are central to social science methods across diverse disciplines, including in feminist critiques of science, technology, and knowledge production (Haraway 1988; Fine 1992; Keller 1995; Harding 2016; Britton and Pritchard 2022), as well as decolonial approaches (Tuck and Yang 2012; Whyte 2018), Indigenous research (Bessarab and Ng’andu 2010; Nelson 2019; Smith et al. 2022), multispecies justice discourse (Celermajer et al. 2020), and more. Here we argue for a need to not only reflect on our values as individual researchers, but also in collaborations, and how we center these within our work. We describe how we have done so and how other research collaboratives might, as well.

As researchers focused on food systems transformation, we perceive strong parallels between what is unsustainable and unjust within food systems, and what is unsustainable and unjust within the academic systems where we work (Lave et al. 2010; Lave 2012; Corbera et al. 2021). For example, both systems focus on increasing productivity to maximize outputs with little regard for matters of equity or diversity (Hicks 2012), or effects on individual livelihoods (Zielke et al. 2022). We are convinced that deep transformation (Meadows 1999) of both systems requires more attention to the values in play within them. The approach we envisage—and have begun experimenting with—is informed by so-called process work (Mindell 2002; Brown 2017). Process work supports diverse groups to surface their shared values, attend to the processes of working together, and discover or forge a shared identity in a fluid way befitting complex, changing environments. Through this work, we have recognized that enacting values we regard as core to sustainability in our research teams and projects is central to our contribution to transformation.

Here, we describe how we have defined collective values and sought to center these in our collaboration as a research collective for sustainability transformations. We explore how we envision centering these values within our research to show how doing so might prevent research into food system transformation from perpetuating ways of working or outcomes which do not align with sustainability ambitions. This specifically requires attention to processes and outcomes of research and the interactions between them (Tengö et al. 2022). We have previously outlined what we identify as values creating harmful academic systems and cultures and what matters to us as a collective seeking a more caring mode of science (Care et al. 2021). In this article, we shift attention to the core focus of our research: food systems and our contributions to transforming them toward sustainability. This continues our attempt to conceive of caring ways of doing research, aligning with others who recognize the importance of not just considering *what* we know but *how* we know and act in the world (Fazey et al. 2020).

In what follows, we elaborate how the values of researchers and research systems can constrain or enable food system transformations research. We describe how a collective of researchers working with a shared ethos of care in personal and professional relations, which we call the Careoperative, have worked to center alternative values sets (Care et al. 2021). We describe our joint definition of core values and then use these as a tool to reflect on two case studies. We close with a discussion of our proposal for affording greater space for emergent transformational outcomes through collectively defining values for research.

## How we conceptualize values

Values refer to what people deem to matter (Rosenberg 2021). While this is subject to change and shaped relationally (West et al. 2018), priorities which guide us can be remarkably persistent. Values are significant because they guide and motivate action and are part of what gives human life purpose or meaning (Kraatz et al. 2020). In sustainability research, values feature in multiple guises with wide-ranging functions, making it vital that researchers reflect on the nature and effects of diverse values (Sharma 2017; Horcea-Milcu et al. 2019).

In this paper, we predominantly consider shared morals and held values as those principles which guide action and help us determine what is right (Chan et al. 2018). We understand values as being a guide (1) for the type of individual researchers we want to be and (2) for the Careoperative’s commitment to transforming food systems. We focus on values associated with “desirable end states or behaviors,” which transcend specific contexts (Schwartz 1992; Horcea-Milcu et al. 2019). However, we acknowledge how values emerge relationally, and become more than abstract notions only through being enacted in specific contexts (Chan et al. 2018). In this spirit, we also recognize shared values as accreting dynamically over time and requiring people’s active participation (Kraatz et al. 2020). The contextual, relational contingency of how values are enacted underlies our perceived importance of actively investigating values in our Careoperative and how these manifest in our research. We are particularly interested in elucidating and describing collective values within our research teams and transdisciplinary projects related to food system transformation for sustainability.

Horcea-Milcu et al. (2019) provided an excellent analysis of four ways in which values have been taken up in such sustainability research. As they outlined, sustainability scientists’ role in relation to values might be to: (1) bring to the surface values which implicitly influence their research, (2) negotiate diverse values feeding into decisions, (3) elicit values ascribed to different elements or choices, and/or (4) focus on values as a leverage point for achieving transformation. Their typology clarified the importance of being explicit and reflexive about values to ensure transdisciplinary work addresses societal needs rather than those of our scientific traditions as researchers. We echo and have sought to enact Horcea-Milcu et al.’s (2019) call for reflexivity in research and to model in our practice values in line with transformational sustainability. Here we hope to contribute to what Horcea-Milcu (2022) notes as under-developed thinking on *how* these aspirations can be engaged and worked with in practice.

## Values-centered approach in our group processes as a research collective

To explore how researchers’ values may be connected to and shape outcomes of research, we first reflect on our journey as a research collective and then on the research in food systems transformations in which we have been involved. Reflexivity and reflection are core inter/transdisciplinary competencies (Bergmann et al. 2005; Popa et al. 2015). Indeed, some definitions of transdisciplinarity make reflexivity core to its purpose (Schauppenlehner-Kloyber and Penker 2015). For example, Jahn et al. (2012) defined transdisciplinarity as, “a critical and self-reflexive research approach that relates societal with scientific problems” (p. 8). Yet, there is a need to further define and develop reflexive, intentional processes that can support the social learning and social experimentation needed to enable sustainable transitions (Popa et al. 2015; Ison et al. 2013). Transdisciplinary integration requires spanning not only cognitive differences but also managing social and emotional aspects (Boix Mansilla et al. 2016; Pohl et al. 2021). There has been relatively little reflexive reporting of researchers' own experience of doing inter/transdisciplinary research (but see Callard et al. 2015). We add to this scholarship by studying ourselves in our group dynamic.

**Fig. 1:**
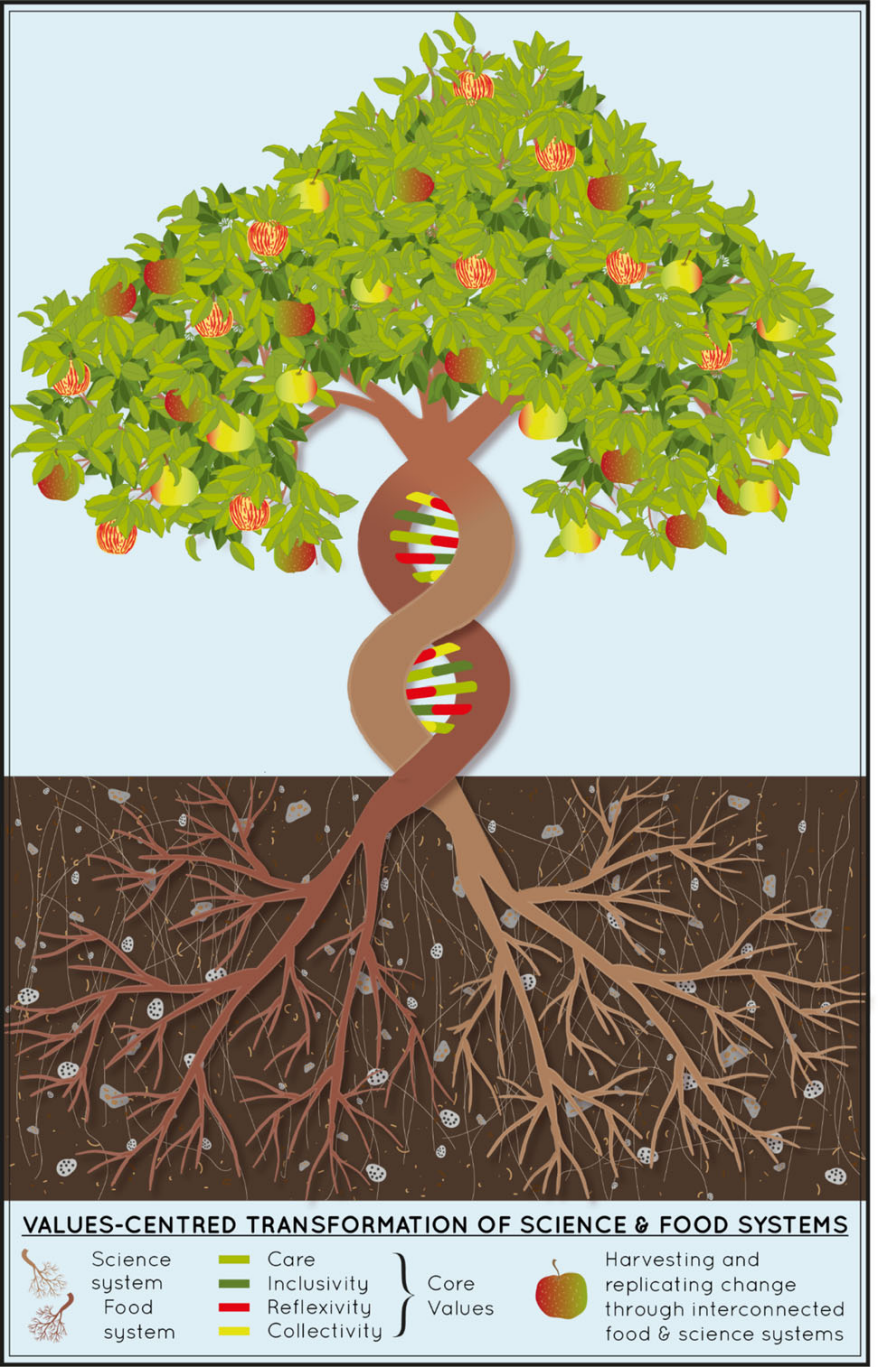
Two tree trunks represent interconnected systems centered on food and academic science. Each has distinct roots, but their need for transformation is parallel. This transformation becomes intertwined when addressed with interacting core values. Through values-centered transformation, the science and food systems grow together and produce fruit and foliage, representing replicating change. By Veronica Remmele

We met as a group of postdoctoral researchers and early-career professors, based at European institutions, within the Postdoc Academy for Transformational Leadership funded by the Robert Bosch Foundation. The two-year program focused on individual leadership. Inspired by each other’s work and the energy within the cohort, we sought to investigate more deeply what “leadership” meant to each of us. We worked through our individual visions and pathways toward a shared definition of what we wanted and how we might get there. Through this work, we formed a research collective in which we intentionally foregrounded our process of working together, rather than our outputs. Our focus on shared values was emergent. We did not start out as values centered in our work together, but became values centered, integrating the four values of care, inclusivity, collectivity, and reflexivity. These values comprise the core of our code of collaboration (Care et al. 2021). In the following, we focus on the emergence of the values that represent and guide our work. We do so to show how these values shape the way we work, learn, research, and interact in service of academic and food systems transformation (see Fig. 1).

In parallel to four official workshops organized by the Postdoc Academy (three in person and one online between 2018 and 2020), we organized a series of our own workshops (between 2019 and 2021) and employed a professional facilitator to work with us throughout. During the first workshop we defined our core values, key elements of how we would work together and named our group "The Careoperative." Our next meeting focused on decision-making, where we learned about and adopted the sociocratic decision-making methods^1^ and developed skills for jointly working in online and hybrid ways. At our third workshop, we learned about dealing with conflict, power dynamics, and managing the emotional side of online working. At the fourth and final workshop, we deepened our immersion in process work. In addition to these four workshops, we held weekly meetings of one-hour, quarterly meetings of half a day, and annual full-day meetings (all online, with occasional hybrid meetings). We also held regional meetings of two to four members, which allowed us to connect and share experiences in person, to complement our online group processes. Across workshops, we sought to refine our horizontal leadership style as well as practice among ourselves these new approaches to working.

Slowing our collective work pace, revisiting and reflecting on what is going well or what needs improving, addressing values, such as inclusivity of all Careoperative members, have been key to our continued collaboration. Inclusivity has been particularly important during phases when members stepped out of core activities due to other responsibilities, including parental leave, new jobs, field work, or leaving academia altogether. A commitment to joint authorship under a collective pseudonym (Dr. Care) is how we attempted to address the ebbs and flows of participation. Trust within the Careoperative has been essential to handling active member dynamics, and enacting values of care, inclusivity, and reflexivity.

Our experience in creating the Careoperative has made a set of values explicit that we now bring to bear in our individual and collective research efforts. Focusing on these values in our common work has led to a working code of collaboration, a dedication to revisiting thoughts on privilege and inclusivity regularly, and rotating, distributed leadership to help us remain collective, caring, and inclusive. Having our work guided by these core values (e.g., on project proposals, shared supervision of students, our collective learning processes, and writing) makes it slower, but allow us to ensure that everyone’s ideas and perceptions are included and negotiated.^2^ While conflicts do arise, we have experience-based trust in our values-centered approach to reaching mutually agreeable outcomes. With the example of our Careoperative, we turn now to exploring the role of (and opportunity to actively shape) values in our individual research spaces.

## Using Careoperative values to reflect on participatory research

We reflect on two illustrative transdisciplinary research projects on food system transformations where Careoperative members were involved and which, in our joint understanding, represent good examples of the challenge of making values explicit in research collaborations. We do so by using our four Careoperative values—inclusivity, care, reflexivity, collectivity—as a lens. First, we present the context and transdisciplinary collaboration for each case study. Then, we use a series of questions to explore how values shaped the research projects and their outcomes: (1) To what extent were care, inclusivity, reflexivity and collectivity embodied in the research?; (2) If the research were repeated, what might be done differently to better incorporate these values?; (3) what is revealed now by reflecting on the cases in light of these values? We note that in the examples, we are careful *not* to impose or confuse the Careoperative values with the values elicited in, for example, the community-led work in Case 2.

### Case 1: Unexplored values lead to a difference in theory of change between Swiss and Malagasy researchers

#### Description

A six-year transdisciplinary research project (2015–2020) on telecoupled landscape changes (Swiss Programme for Research on Global Issues for Development, 2023) to place in three countries, Madagascar, Laos, and Myanmar, through a research partnership between researchers from Switzerland, and National Universities or civil society organizations in the three countries. Here, I (Julie G. Zaehringer) focus my reflection on the case of the Swiss-Malagasy research collaboration.

The Swiss and the Malagasy researchers worked together in north-eastern Madagascar, where shifting cultivation (or slash-and-burn agriculture) for subsistence rice production has been the leading cause of deforestation, although not the only one (Llopis et al., 2019; Zaehringer et al., 2015). The project’s goal was to co-design innovative approaches for more sustainable land use and land governance to promote co-benefits between ecosystem service supply and human well-being. This was addressed through three distinct phases: a transdisciplinary pilot phase to adapt research questions to the local context; an empirical phase to collect data through interdisciplinary approaches on the same village case studies; and an implementation phase to co-design and pilot small experimental implementation projects based on the empirical findings together with stakeholders.

We, the Swiss researchers in the project, had already been working on shifting cultivation previously (Messerli, 2004; Zaehringer et al., 2015, 2016). Through being active in global scholarly communities and debates on the future of shifting cultivation, we perceived it as a well-adapted and valuable land use system. Our normative stance was that shifting cultivation should be allowed to thrive, as long as the fallow length would be sufficient to replenish nutrients, and expansion into remaining forests could be avoided. Several years into the project, during a stakeholder workshop, a Malagasy colleague in the project described their stated goal as, in contrast, aiming to eradicate shifting cultivation regimes. Only at this point did it become clear that assumptions about shifting cultivation differed between our research teams, which might have influenced how research results on land use changes were interpreted and discussed with stakeholders. For example, representatives of conservation organizations, part of the stakeholder process, might have taken this as confirmation for interventions trying to replace shifting cultivation through permanent agricultural practices, while representatives of farmer organizations might have used this evidence to support their claims on land taken by the National Park.

#### Reflection with Careoperative core values

The research project described here was already built upon and showcases numerous core values, including, for example, **inclusivity** and **collectivity**. These values were prioritized during the project design, as the project aimed to apply a decolonial research approach: for example, the head of the Malagasy research team was also a Co-Principal Investigator in the overall research project and the budget allocated to the Malagasy team was higher in absolute terms than the budget to hire researchers in Switzerland. But a more *intentional* and *explicit* centering of values, e.g., through a facilitated discussion regarding sustainability issues in the landscape, could have strengthened these values of inclusivity and collectivity. This would have highlighted the need for additional **reflexivity** on the topic of shifting cultivation in the wider team at an earlier stage.

Instead of first gauging our own values, as well as those of Malagasy colleagues on this crucial topic, we directly delved into social learning, convening a regional group of nonacademic forest and agriculture stakeholders. Assuming that everyone in the project was working on equal terms, we inadvertently ignored how cultural norms shape assumptions of any sustainable development transformation issue (le Polain de Waroux et al. 2021). Moreover, a strong sense of collegiality or even friendship had evolved within the team, emphasizing the value of **care**, which created a certain discomfort in addressing fundamental differences in values. Here we see the ways that different values also interact. While **care** was important to the project, when not coupled with sufficient **reflexivity** it created a hurdle to engaging with different views.

A value-centered approach could have led to prioritizing a discussion about what individual views regarding sustainable land use prevailed and how these might be influenced by disciplinary and cultural backgrounds. While consensus might have been elusive, either within the research team or between the stakeholders, **reflexivity** around different views on shifting cultivation could have afforded the team a way to more clearly understand the implications of decisions regarding land use. Values such as **inclusivity**, **collectivity** and **care** were already guiding the project design and implementation. A more explicit approach to joint **reflection** on the values held by different researchers and about how these were influencing the research process and our collaboration might have had a higher likelihood of helping the project realize its transformative ambitions. In our experience, it is common with many collaborative research projects that consensus and alignment of values among different researchers in a team are presumed from the onset, rather than openly explored and reflected upon at the start of the project. In North–South research partnerships, such as the one in the example, this can be further hampered as Northern researchers might fear that if they propose such an exercise, the assumption that values differ between team members might be perceived as an “othering” process and therefore reinforcing colonial structures instead of tearing them down.

### Case 2: Contrasting values emerge from a cooking workshop in Tajikistan

#### Description

I (Jamila Haider) have been working with Tajik collaborators since 2009 to understand relationships between biological and cultural diversity in the Pamir region, Tajikistan. While the Pamir region remains one of the poorest post-Soviet areas, it is also an area of high biocultural diversity, and the center of origin for many fruit, nut, and grain varieties (Vavilov 1917). Working first as a development practitioner in the region (2009–2011) and then as a researcher using resilience assessments (Haider et al. 2012), I engaged in activities of envisioning future development pathways. These workshops were often dominated by prominent men from the communities we worked with. When we asked “what do you wish for the future” responses included: improved seed varieties, fertilizers, and improved access to the markets, characterized by individualist and profit-driven values.

Hospitality is an important value in the Pamirs, and we were often served food, predominantly meat-based ‘Russian’ dishes. One day, we served an incredible vegetarian noodle soup (*osh*) where the noodles are made of at least six different grains and legumes, which are also cultivated together. The grandmother who served the soup asked us to write down the recipe, since Pamiri languages are unwritten and she said that her children and grandchildren did not care much for the old traditions. With Pamiri collaborators, we began to collect recipes, culminating in the publication of a Pamiri recipe book (van Oudenhoven and Haider 2015). Using cooking as participatory methodology elicited emotions, ideas and values that contributed to alternative visions for the future to the ones I had heard in previous workshops (Haider and van Oudenhoven 2018). In 2016, we co-organized (with communities and the NGO) a food celebration day, where different communities from across the Pamirs brought specific local food products to cook with. Throughout two days different groups set up cooking stations outside, we met periodically inside homes to capture stories through drawing, and over time stories emerged from the cooking sessions, presenting a range of values and visions. For example: *this food makes us strong, whereas the food we buy from the shops makes us sick*; or, *the importance of reciprocity in times of need and how reciprocal acts are not necessarily equal but rather equitable*. A watercolor artist was present during the cooking workshops and captured some of these values and ideas in the form of four different future visions.

**Fig. 2:**
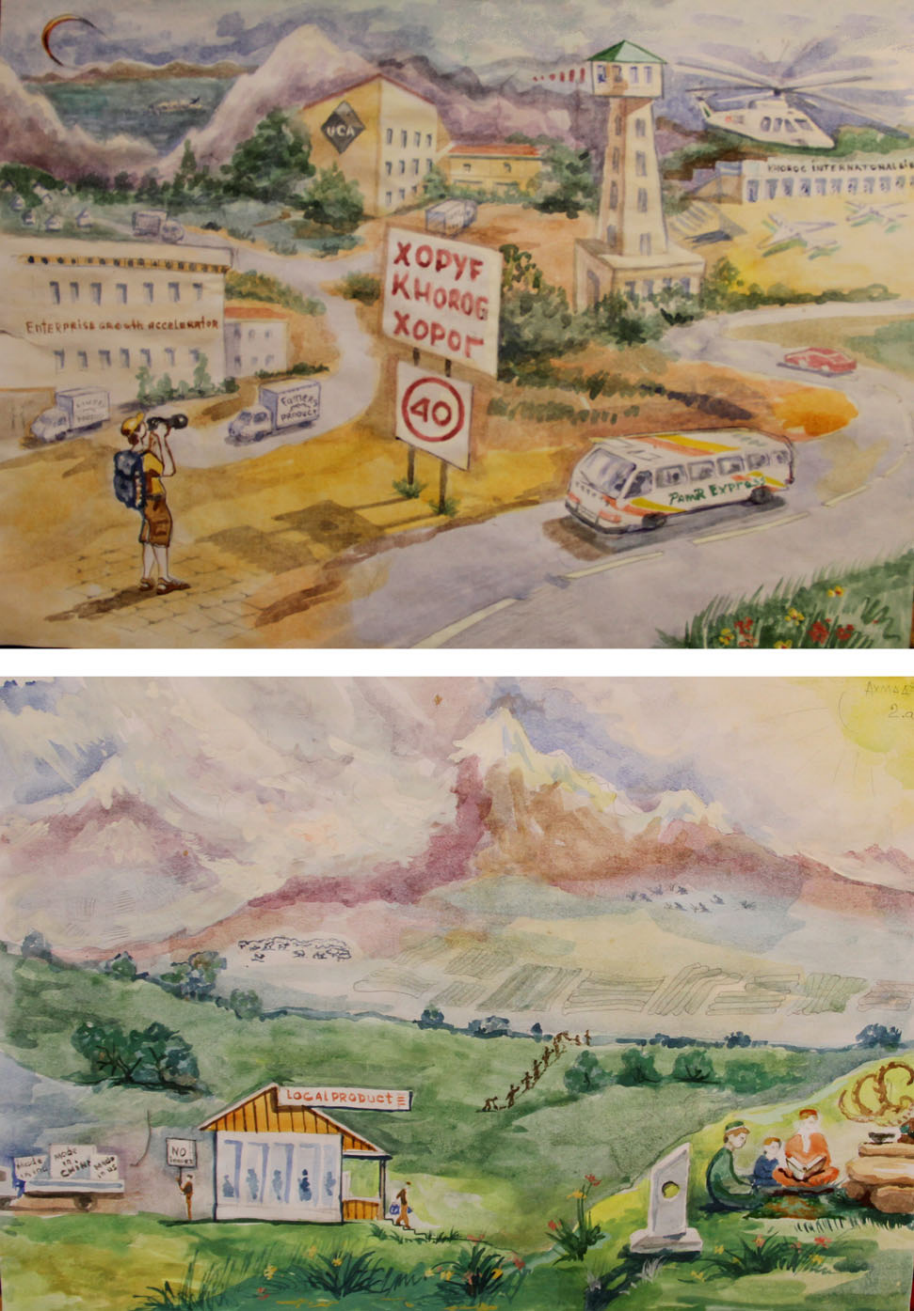
Two different visions of the future in the Pamir mountains. In the top illustration, the NGO vision of development is depicted: high-speed bus on motorway and international airport facilitating tourist access, an ‘Enterprise Growth Accelerator’ and international university. The bottom illustration features a community vision of food sovereignty. A local shop is set up, with a truck full of imported food being turned away. A family shares knowledge next to a shrine and traditional sundial. In the background, people engage in collective work to repair an irrigation channel and engage in rainfed agriculture to adapt to climate change. **A** previously published in (Haider 2017) and **B** previously published in (Haider & Cleaver 2023). Both used with permission. Water color artist Yorali Berdov

The NGO had a strong vision of increased productivity and growth in the region (Fig. 2). In contrast, the second vision of Fig. 2 from a Village Technology Group is about food sovereignty and therefore not having any imports, while also trying to increase rainfed agriculture so as not to involve irrigation. The other two community groups had values at the forefront of their visions. Hospitality and intergenerational knowledge exchange plus better livelihood opportunities were central to their visions, voicing their desire to maintain these important values.

#### Reflection with Careoperative core values

The cooking workshops were not designed to elicit values, but rather were designed in an open way, to be inclusive and embrace surprise. In reflecting what could have been different with a Careoperative-centered values approach, the author observed how the open, participatory, material (i.e., food), embodied (i.e., cooking) foci of the research already expressed the values of inclusivity, care and collectivity. In this case, the preparation of food (generally done by women whose voices are often marginalized in this context) explicitly elevates sharing, hospitality, and inclusion, which contributed to a celebration of otherwise under-appreciated knowledge and skills. What this case reveals is that an open, participatory process enabled resonance between the values of the research team and the values of the community involved in co-production. The values centered by the communities, sharing and hospitality, in conjunction with the researcher’s explicit value of subverting power dynamics and marginalization of knowledge, were central to the design of the food celebration workshops. Reflecting on these in turn reveals how inclusivity, collectivity, caring, and reflexivity were embedded throughout the process.

NGO and community groups were **included** in the design of the visioning workshop, and a local artist was involved in all steps. Invitations were open for community members and through cooking as participatory methodology, more marginalized groups in Tajik society, such as women and children, were centrally involved. Since food is emotive and evocative, it is a way to break-down traditional power dynamics and hierarchies (Haider and van Oudenhoven 2018). Further, the point of the work was to elicit difference through visions, rather than consensus. **Collective** cooking and eating are common in Pamiri culture and were therefore a natural way to collectively come together to envision futures. In this context in Tajikistan, coming together around food is a way to elevate marginalized voices (of women). Preparing and celebrating food together are also a way of **caring**, involving elderly people and children and explicit recognition and gratitude to the material world from which food comes. The artist played a key role in the **reflexivity** of the project, since the visions through water color paintings were shared and discussed among communities and the NGO and researchers from the local university.

## Discussion: emergent, value-centered research on food system transformation

We have sought to illustrate the implications of stepping toward being values-led transformational researchers. The two cases presented different expressions of **inclusion, caring, reflexivity, and collectivity** playing out in different ways. By centering the values at play *in each collaboration*, rather than *of various research subjects*, our work represents a reflexive dimension of the four foci that Horcea-Milcu et al. (2019) suggested. Specifically, centering values provides an invitation for research teams to critically reflect on the values that influence their research; negotiate diverse values when working together; elicit the repercussions of centering different values in collaboration; and focus on values related to how to work together that resonate with, for example, research goals aligned with food system transformation. As researchers engaged in food system transformations, engaging with nonacademic actors, our academic affiliation comes with power, which is accompanied with the responsibility of making values explicit (Boyce et al. 2022). Our aim in this contribution has thus been to foreground possible questions about how to design collaborative research to more systematically and intentionally reflect values aligned with transformational sustainability outcomes.

The two juxtaposed case studies highlight how the framing of research can influence which values are made explicit and which remain implicit. In each case, the materiality of the research topic served as a focal point for embodiment, expression, and exploration of values (of the researchers and in the substance of the research). In Case 1, the materiality of landscape management practices surfaced different values held by people within the research team itself. An earlier discussion regarding sustainable land use, based on values of inclusivity and reflexivity, may have surfaced different values earlier, allowing for subsequent activities to proceed differently. In Case 2, the materiality of food and cooking afforded a method to elevate women’s knowledge and create space for a plurality of values within the community to be expressed. The seemingly simple act of giving women, otherwise marginalized in the community and relegated to the kitchen, a spotlight on the food they bring into the world and their knowledge of doing so let them shine in a way that elevated their voices.

The contrasting nature of the two projects may suggest that different kinds of research more easily lend themselves to centering values, but we suggest that all transformational researchers can benefit from reflecting on the role and potential of values in their work. From the first case, we observed how a values-centered approach can surface not only important information about ways of working together but also content issues of vital importance to transdisciplinary research, such as ways of valuing different approaches to shifting cultivation. From the second case we saw how values of inclusivity, collectivity, care, and reflexivity were implicitly embedded in open, participatory, co-production research.

Going further, the two projects offered examples of contrasting structures, temporalities, and approaches as they play out in projects. Case 1 illustrated a large diverse international research consortium working toward a somewhat proscribed goal. Case 2 featured a single, case-based study with one main researcher using long-term ethnography of a more exploratory tenor. Upon reflection, we can see how important it could be to *a-priori* elicit and articulate values among researchers and partners in a large inter-cultural, inter- and transdisciplinary consortium. In contrast, the fluid open space created by the individual ethnographic researcher was conducive to eliciting values in situ and as part of the research process, where in this case Pamiri values of hospitality and sharing resonate strongly with inclusivity, collectivity, and care.

There are other more quotidian ways in which researchers can take values-centered approaches into their work. We previously suggested that part of our collective leadership work is to ‘seed’ what we have learned and experienced through the Careoperative within other contexts where we work individually or as smaller sub-groups (Care et al. 2021). Acknowledging existing hegemonic power structures within academia, we subscribe to a theory of change in which a fractal of actions (O’Brien et al. 2023) unfold across the academic landscape, changing not all at once or overnight, but inexorably, non-linearly, over time.

In this way, we have dispersed our core values into other projects and groups and applied aspects of our process work in new settings. For example, some of us encourage PhD students we supervise to include reflexivity more fully within their research. Others who have leadership roles have been able to build teams founded in inclusivity and care, selecting team members in light of these values rather than purely on the basis of traditional criteria of academic excellence. As managers, we dedicate time with teams for checking-in and reflecting on how the group is working toward its shared values. Many of us have recommended specific techniques such as sociocratic decision-making and participatory budgeting within our workplaces, so as to bend structures and systems typically dominated by less transformational values. Other seeds sown from our core values are less overt, such as being more active listeners and seeking a more caring dynamic to our workplace interactions.

To counter systemic obstacles to adoption of these values, we have worked to create caring, reflexive, inclusive sessions—seeking to draw attention to these obstacles—in international conferences.^3^ Another fundamental choice we make is in what we work on. For example, choosing to undertake co-productive research with marginalized groups (e.g., Lazurko et al. 2023); choosing to take on projects to reform funding programs and research cultures (e.g., Smith et al. 2023); choosing to research the research process to identify step-change improvements in transformative research practice (e.g., the FOSTER project (https://fosterfoodsystem.eu/). Choice also extends to the courses we teach; for example introducing transformative teaching through methods schools for faculty; integrating embodiment methods into courses (e.g., through a bike excursion to lived utopias); equipping professionals with transformative leadership skills and sustainability.^4^ In these ways, our ongoing involvement with the Careoperative flavors our daily work and relationships, while our collective reflexivity provides space to explore how we can go further, or the challenges we face in doing so, not least the limits of our power to make change.

As we have discussed elsewhere (Care et al. 2021), we realize that these ideals are not always possible in practice, particularly in an environment of many constraints on time and funding, and pressure to chase high impact factors. The ease with which one reads the list of examples above glosses over the lived, day-to-day struggle of expressing these values in practice. A range of structural factors related to funding institutions and research organizations are implicated by our reflections. As Smith et al. (2022) observed from working with a European Research Area network program to better align values of responsible innovation and sustainability with research programming and project work, transforming research culture benefits from a focus not only on resources, but also relationships, institutions, and types of expertise included. Further, we are conscious that it may be easier to focus on processes and shared values within homogeneous groups. However, assuming homogeneity of a group based, e.g., on gender, ethnicity, or discipline may lead to assumptions of greater similarity than actually present. This can create less space for disagreement to surface and be productively integrated into the group process. Group composition thus presents challenges around inclusivity, but also the opportunity to reflect on whether collaborative echo chambers may be limiting transformation. Observing this suggests the need to bring the critical awareness of values in collaboration to the fore to allow project teams to hold pluralistic, sometimes incommensurable perspectives in productive tension.

## Conclusions and outlook

To develop our thinking and practice as researchers, we reflected on two case studies, centering values of inclusion, caring, reflexivity, and collectivity. Bringing such values to the fore can better support an alignment of the sustainability transformations needed in food systems and food systems research. We do not suggest that inclusion, reflexivity, care, and collectivity are values all projects must adopt. Rather, we suggest there is, qualitatively and substantively, a different experience and outcome from working in teams where values of process and values of project goals resonate (qua Rosa 2016). As McGreevy et al (2022) observed, principles for sustainable food systems will require new relational, allocative, and institutional principles, which we argue also requires different ways of working together on such food system transformations.

Based on our experiences above and in the Careoperative (Care et al. 2021), a range of concrete lessons are helpful to consider for taking forward at levels of group collaboration:

- Surfacing and holding: Being clear in advance about centering values related to collaboration, surfacing different values within the group, and holding space to address self/group differences as well as tensions (e.g., over power, position, relationships, etc.). Particularly vital here is to make explicit the values of collaboration that may be implicit, and to hold space for a plurality of values.
- Learning and practicing: Co-developing skills for working together through conflicts, as they arise, in productive ways (e.g., resistance-based consensus processes that proactively surface and address disagreements; active, non−judgemental, curiosity-based-as opposed to hierarchical inquiry to discover and pragmatically address differences, c.f., Kühn 2021). Such efforts benefit from the support of specific sensibilities and capacities, skilled expertise, and facilitation resources.
- Pausing and reflecting: Embracing surprise and responding through trial and error; reflecting periodically on process; attending with care to relationships and interactions.

Apparent across these three stages is that there is always potential for conflict or tensions around divergent values and how they might be negotiated or resolved. We are highly aware that for us this was less problematic than it might be in other cases, our being a relatively homogenous group—predominantly female, white, early- to mid-career and from Minority World countries.^5^ We also recognize our fortunate positions of having the flexibility and degree of autonomy necessary to facilitate inclusive, reflexive conversations. We have prioritized these generative and long-term relationship-building activities, and the time they require, despite academic institutional contexts' tendency to view them as unproductive. Such tensions might be more apparent, extreme, and complex within more diverse collectives or when two such collectives attempt to collaborate. In the case of the Careoperative, we have recognized the co-equal importance and inextricable interconnectedness of practicing with values of inclusion, care, reflexivity, and collectivity. Another group of potential collaborators may have values more geared to—borrowing an academic stereotype—“publish or perish,” which entails the prioritization of publication production. Here, the question to ask is not whether one value set is “better” or “more important” than the other, but rather can the two groups arrive at a mutually agreeable way to operate together and achieve a common goal. In our experience, adopting a process work approach offers one way to navigate such a negotiation.

A further empirical question arises for sustainability transformation research: are certain sets of values in research more conducive to sustainability transformation than others? In light of this uncertainty, it is paramount to surface latent values and attend to the differences they make in research practices. As a first step, research designs would benefit from creating space for exploring researcher’s values, along with those of other actors involved in knowledge co-production. Second, once values are made explicit, it might become possible to question them and trial new ones, along with corresponding practices. In this sense, attending to our research practices, funding, academic systems, and networks offers a deep leverage point for sustainable food systems transformation.

## Footnotes

1 https://sociocracy30.org.

2 Which is symptomatic of conducting this work in the margins of each of our “day jobs.”

3 For example, through a session using forum theater to explore values of care, inclusion, collectivity, and reflexivity in academe; to create spaces within the conference that express these values—lactation rooms for nursing mothers; accessibility by design; addressing these values in the program explicitly. PECS 2024 Conference https://event.fourwaves.com/pecs2024/pages.

4 https://uit.no/utdanning/program/718168/ocean_leadership_-_executive_master.

5 Minority World is a term used to disrupt predominant ways of talking about “developed” and “developing” countries, reflecting global population numbers, instead. See for example Khan et al. 2022 and https://www.npr.org/sections/goatsandsoda/2015/01/04/372684438/if-you-shouldnt-call-it-the-third-world-what-should-you-call-it.
